# Supplementary material for: The therapeutic effect of glycyrrhizic acid compound ointment on imiquimod-induced psoriasis-like disease in mice
Source: PLoS One. 2023 Aug 29;18(8):e0290637. doi: 10.1371/journal.pone.0290637 (PMC10464954; doi:10.1371/journal.pone.0290637)
Supplement: Attachment — Submitted filename: Responds to the reviewers comment.docx [file pone.0290637.s001.docx]

Dear reviewer,

Thank you very much for your comments and professional advice. These opinions help to improve academic rigor of our article. Based on your suggestion and request, we have made corrected modifications on the revised manuscript. We hope that our work can be improved again. Furthermore, we would like to show the details as follows:

**Reviewer #1**

Comment: New, safer treatments for psoriasis are needed. This manuscript provides a new approach that could also be safe and readily available. The experiments are well done, statistically valid and potentially clinically important. The English can be improved.

**Response:** Thank you for this valuable feedback, and the whole manuscript has been polished accordingly.

**Reviewer #2**

**Comment 1:** However, a quick literature review {34044769; 27786567; 26629143} showed studies presenting similar idea that glycyrrhizin improves psoriasis. I believe it’s crucial the authors need to more clearly explain the novel addition of their study to the knowledge we already have regarding glycyrrhizin therapeutic effects.

**Response:** Although the antipsoriatic effects of glycyrrhizic acid have been similarly reported, our paper was optimized for the associative properties of glycyrrhizic acid and other natural drug monomers. It was the most potent natural drug monomer blend ointment reported to date, and it achieved the potency of calcipotriol, which had not been demonstrated by any other method before. And unlike other studies, we used transdermal administration rather than oral administration, which significantly reduced the dose administered. It was an important addition to glycopyrrolate for psoriasis treatment.

At the same time, we found no reduction in spleen weight in psoriatic mice with calcipotriol ointment. It was reported that calcipotriol induced atopic dermatitis-like lesions in mice, so we questioned the use of calcipotriol as a positive drug in a mouse psoriasis model. We show this experimental phenomenon of calcipotriol as a positive drug when studying psoriasis and hope that other investigators will take this into account in future studies.

We thank the reviewer for this comment and realize that these differences may not have been expressed clearly enough in the previous manuscript. We have improved the original manuscript, particularly in the introduction (paragraph 2), to clearly illustrate the differences in this study.

**Comment 2:** The study was carried out on female mice only. Is there a specific reason for that? Are male mice going to be studied later on?

**Response:** Thanks for reviewer’s comment, the study by Pilar Alvarez et al. [1] showed that Female mice develop more severe disease than male mice in response to generic imiquimod cream. Meanwhile, in the course of our experiments, we found that male mice induced with imiquimod showed mostly no skin damage. With regard to gender differences in psoriasis models, this is something we need to work on in the future and we are working on this, and you will see research in this area in our future work.

[1]Pilar Alvarez, Liselotte E. Jensen, "Imiquimod Treatment Causes Systemic Disease in Mice Resembling Generalized Pustular Psoriasis in an IL-1 and IL-36 Dependent Manner", Mediators of Inflammation, vol. 2016, Article ID 6756138, 10 pages, 2016. https://doi.org/10.1155/2016/6756138

**Comment 3:** In the H&E experiments, fig.3, How was the epidermal thickness determined? How were such results normalized with regard to normalization pixelated images, number of samples/sections per mice?

**Response:** We apologize for the omission of the description of the skin measurement method, the relevant method has been added to the Materials and Methods section:

“All fields of view of each H&E-stained section were photographed continuously (200× magnification) using natural light sources with manual exposure. HE sections were analyzed using Image Pro-plus6.0 software (IPP, Media Cybernetics, USA). Calibration of optical density and scale bar. Echinoderm thickness measurement: the anterior, middle and posterior fields of view of each section were measured, and the thickness of the echinoderm was measured at five different random locations in each field of view.”

**Comment 4:** In the discussion section, it was stated that the current hypothesis for psoriasis pathogenesis is through the activation of T cells. I believe more references are needed to support this hypothesis.

**Response:** We are very sorry for our careless mistake and we have added references in the discussion section:

“[21]Coimbra S, Figueiredo A, Castro E, Rocha-Pereira P, Santos-Silva A. The roles of cells and cytokines in the pathogenesis of psoriasis. Int J Dermatol. 2012;51: 389–398. doi:10.1111/j.1365-4632.2011.05154.x”

**Comment 5:** Statement of funding is not complete. In the discussion section: paragraph 2, first line: check for spelling.

**Response:** We are very sorry for our careless mistake and we have fixed the spelling mistake of "reduced". Meanwhile, we have added the fund information to the online submission form.

Thank you very much for your attention and time . Looking forward to hearing from you.

Yours sincerely,

Shuangyong Sun

18 May., 2023

Tianjin Medical University, No. 22 Meteorological Station Road, Heping District

300041 Tianjin China;

Tell: 18622560939; Fax: (022)84180630;

E-mail addresses: 1428004481@qq.com
